# Reported Radiation Overexposure Accidents Worldwide, 1980-2013: A Systematic Review

**DOI:** 10.1371/journal.pone.0118709

**Published:** 2015-03-19

**Authors:** Karen Coeytaux, Eric Bey, Doran Christensen, Erik S. Glassman, Becky Murdock, Christelle Doucet

**Affiliations:** 1 Episight Consulting, Summit, New Jersey, United States of America; 2 Plastic and Reconstructive Surgery Department, Percy Military Hospital, Clamart, France; 3 Radiation Emergency Assistance Center/Training Site (REAC/TS), Oak Ridge, Tennessee, United States of America; 4 Celogos, Paris, France; Institute for Health & the Environment, UNITED STATES

## Abstract

**Background:**

Radiation overexposure accidents are rare but can have severe long-term health consequences. Although underreporting can be an issue, some extensive literature reviews of reported radiation overexposures have been performed and constitute a sound basis for conclusions on general trends. Building further on this work, we performed a systematic review that completes previous reviews and provides new information on characteristics and trends of reported radiation accidents.

**Methods:**

We searched publications and reports from MEDLINE, EMBASE, the International Atomic Energy Agency, the International Radiation Protection Association, the United Nations Scientific Committee on the Effects of Atomic Radiation, the United States Nuclear Regulatory Commission, and the Radiation Emergency Assistance Center/Training Site radiation accident registry over 1980-2013. We retrieved the reported overexposure cases, systematically extracted selected information, and performed a descriptive analysis.

**Results:**

297 out of 5189 publications and reports and 194 records from the REAC/TS registry met our eligibility criteria. From these, 634 reported radiation accidents were retrieved, involving 2390 overexposed people, of whom 190 died from their overexposure. The number of reported cases has decreased for all types of radiation use, but the medical one. 64% of retrieved overexposure cases occurred with the use of radiation therapy and fluoroscopy. Additionally, the types of reported accidents differed significantly across regions.

**Conclusions:**

This review provides an updated and broader view of reported radiation overexposures. It suggests an overall decline in reported radiation overexposures over 1980-2013. The greatest share of reported overexposures occurred in the medical fields using radiation therapy and fluoroscopy; this larger number of reported overexposures accidents indicates the potential need for enhanced quality assurance programs. Our data also highlights variations in characteristics of reported accidents by region. The main limitation of this study is the likely underreporting of radiation overexposures. Ensuring a comprehensive monitoring and reporting of radiation overexposures is paramount to inform and tailor prevention interventions to local needs.

## Introduction

Radiation overexposure accidents are uncommon, but can have severe long-term health consequences. Radiation is used in various settings. Major sectors include industrial, medical, and military. Some key applications are electricity production, sterilization of material equipment or food, development of nuclear weapons, radiography imaging techniques for welds inspection, radiation therapy, and radiology imaging techniques (e.g. X-ray radiography, fluoroscopy, computed tomography). These last decades, the medical sector in particular experienced a fast growth in the use of ionizing radiation that allowed better diagnostics and treatments [1–2]. In order to guide all facets of prevention, it is critical to understand the reasons and events behind radiation overexposures.

Radiation-related harms have been reported over the years in all sectors, along with those resulting from orphan sources. Harmful effects of radiation overexposure include deterministic effects (e.g. radiation sickness, skin radiation burn, cataracts, infertility) and stochastic effects (e.g. cancer). These effects can take from weeks to years to manifest, with severity depending upon multiple parameters including the total radiation absorbed dose, the radiation dose rate, the volume of body exposed, the parts of the body and tissues involved, the radiation source at stake, as well as personal characteristics of overexposed people (e.g. age, health status) [3].

Furthermore, local and global overexposures to the body translate into different health outcomes and therefore different treatment needs. Global overexposure of 1 Gray (Gy) or above induces acute radiation syndrome (ARS) characterized by consecutive hematopoietic, gastrointestinal, and neurovascular syndromes [3]. Local skin overexposures of 3 Gy or more are likely to lead to acute local radiation injuries (LRI), which may be associated with extreme pain. Local organ overexposures of typically 2–8 Gy are likely to result in organ dysfunction (e.g., permanent sterility of ovaries and testes, acute pneumonitis, renal failure, cognitive defect) [4]. In addition, this type of injuries often progresses over time due to inflammatory waves, inducing the spread of radionecrosis, and requires long-term treatment [5].

In order to decrease the risk of harm associated with ionizing radiation, its use is often regulated at the country level. At the international level, the United Nations Scientific Committee on the Effects of Atomic Radiation (UNSCEAR), the International Atomic Energy Agency (IAEA), and the International Commission on Radiological Protection (ICRP) play a central role. They evaluate radiation risks, provide recommendations, and promote safe use of radiation technologies, which evolve rapidly and gain in complexity. Yet, in an era where resources are scarce, it is essential to identify the most pressing issues in order to better target prevention actions such as training, which can lead to dramatic improvements in the safe use of radiation [6].

Several non-systematic reviews of radiation overexposure accidents have been published in the literature. The UNSCEAR organization performed the most extensive review, which it considers "a sound basis for conclusions regarding the number of significant radiation accidents that have occurred, the corresponding levels of radiation exposures and number of deaths and injuries, and the general trends for various practices", despite unavoidable underreporting [7]. Additionally, several voluntary registries of radiation overexposures have been developed. The Radiation Emergency Assistance Center/Training Site (REAC/TS) at the US Department of Energy's (DOE) Oak Ridge Institute for Science and Education (ORISE) maintains the largest US national and international radiation accident registry to our knowledge [8]. Other registries focus only on the medical sector. For example, the Radiation Oncology Safety Information System (ROSIS) and Safety in Radiation Oncology (SAFRON) register incidents and near incidents in radiation therapy [9–10].

To build further on the existing work and provide new information on characteristics and trends of reported radiation accidents worldwide, we performed a systematic review of reports published between 1980 and 2013 by querying the peer-reviewed literature, national and international reports, and the REAC/TS registry. Our objective was to evaluate the impact of past prevention programs and potential remaining needs for prevention planning. In this report, we present the search strategy and results of our review, and a descriptive analysis of the retrieved radiation overexposure accidents.

## Methods

### Sources

A protocol was developed for the conduct of this systematic review and is detailed in S1 PRISMA Checklist and S1 Protocol.

Reported radiation overexposure accidents from 1980 to present were searched using MEDLINE, EMBASE, the IAEA publications, the congress proceedings of the International Radiation Protection Association (IRPA), the collection of UNSCEAR reports, the United States Nuclear Regulatory Commission (US NRC) reports on abnormal occurrences, and the REAC/TS registry.

For the purposes of this review, we elected to use the IAEA definition of accident, which is "Any unintended event, including operating errors, equipment failures or other mishaps, the consequences or potential consequences of which are not negligible from the point of view of protection or safety" [11]. Our study focused on radiation accidents resulting in one or several people overexposed and meeting our inclusion and exclusion criteria.

### Search strategy

We conducted an electronic search in MEDLINE and EMBASE on March 27th 2014 for all relevant articles published since January 1^st^ 1980, in the English or French languages. The MEDLINE search strategy included a first keyword search in titles using: cause of overexposure (radiation, nuclear) AND type of injury (overexpos*, accident*, injur*).

A second keyword search in titles was performed as follows: cause of interventional radiology overexposure (fluoroscop*, diagnostic, imaging, angioplast*, catheter*, coronar*, arter*, endovasc*, cardiac, stent*, cardiol*, shunt*) AND type of injury (radiodermatitis, "radiation dermatitis", radionecrosis, "radiation necrosis", "radiation injury", "radiation injuries", "radiation effect", "radiation effects", erythem*, "radiation-induced skin", “skin injury", “skin injuries", ulceration) NOT (erythematosus or lupus).

The third search was based on the following Mesh terms: (("Radiodermatitis"[Mesh] OR "Acute Radiation Syndrome"[Mesh] OR ("Radiation Injuries/mortality"[Mesh] OR "Radiation Injuries/radiation effects"[Mesh] OR "Radiation Injuries/radiography"[Mesh] OR "Radiation Injuries/radionuclide imaging"[Mesh] OR "Radiation Injuries/radiotherapy"[Mesh]) OR ("Radiotherapy, Computer-Assisted/adverse effects"[Mesh] OR "Radiotherapy, Computer-Assisted/mortality"[Mesh]) OR "Radiotherapy, Image-Guided"[Mesh] OR "Whole-Body Irradiation/adverse effects"[Mesh] OR "Fluoroscopy/adverse effects"[Mesh])) AND ("case reports"[Publication Type] OR "review"[Publication Type]) AND "humans"[Mesh].

The EMBASE search strategy used the following words of major interest: 'radiation accident'/exp/mj OR 'radiation injury'/exp/mj OR 'acute radiation syndrome'/exp/mj OR 'radiation dermatitis'/exp/mj OR 'radiation sickness'/exp/mj OR 'radiation necrosis'/exp/mj AND [humans]/lim AND ([english]/lim OR [french]/lim) AND [1980–2014]/py AND [embase]/lim NOT ([medline]/lim AND [1980–2014]/py OR 'radiation recall' OR 'pneumonitis'/exp/mj OR 'nephrititis' OR 'efficacy' OR 'trial' OR 'sodium arsenite'/exp/mj OR 'neurofibromatosis'/exp/mj OR 'cisplatin'/exp/mj OR 'vinorelbine'/exp/mj OR 'enteritis'/exp/mj OR 'experimental' OR 'bevacizumab'/exp/mj OR 'chemoradiotherapy'/exp/mj OR 'simvastatin'/exp/mj OR 'cetuximab'/exp/mj OR 'cefotetan'/exp/mj OR 'gemcitabine'/exp/mj).

Reference titles and summaries were screened manually and discarded if not relevant. Selected publications were read in full text for data extraction. Cross-referencing was used to retrieve additional relevant articles.

The IAEA publications (nuclear safety reviews, safety reports series, and non serial publications on radiological accidents from 1980 to 2013), the IRPA congress proceedings(1980–2008), the UNSCEAR reports (1980–2013) and the US NRC reports to congress on abnormal occurrences (1980–2012) were systematically read in full text and extracted when relevant.

Finally, radiation overexposure accidents that occurred on or after January 1^st^, 1980 and reported in the REAC/TS registry were retrieved using filters against our inclusion criteria. Full reports were read for retrieved cases and extracted if relevant.

### Inclusion and exclusion criteria

A case of radiation overexposure was defined as presenting at least one of the following criteria: (i) unintended global overexposure of 1 Gy or more, (ii) unintended local skin overexposure of 3 Gy or more, (iii) unintended local organ overexposure (e.g. brain, thyroid, prostate) of 5 Gy or more, or (iv) description of clinical presentation providing reasonable index of suspicion for unintended ionizing radiation overexposure (i.e., acute radiation syndrome, radiodermatitis, permanent alopecia, dry or moist desquamation, blister formation, skin ulceration, dermal atrophy, invasive fibrosis, organ failure, radio-necrosis). The thresholds used in this review are based on the literature, keeping in mind that these thresholds are not absolute boundaries [3], [4], [12–18]. Cases that met none of these criteria were excluded, as were suicide and criminal acts.

For cases without occurrence date, we used date of first symptoms as first proxy and date of report as second proxy.

Finally, similar cases issued from different reports but with insufficient information to decipher whether they were different or not, were not integrated in the database to prevent duplicates.

### Selection process

Two independent researchers screened and reviewed data sources against the inclusion criteria. For selected reports, full-text documents were evaluated and extracted manually by one reviewer and double-checked by a second reviewer. Any divergence between reviewers regarding selection process was resolved through discussion.

### Extracted data items

For each accident, select information was extracted into a data sheet. Selected data included date and place of occurrence, number of overexposed people and number of people dying from their overexposure, days between exposure and death, type of overexposed people (i.e., patient, public, or worker if dose was received in the course of employment), estimated global and local dose received, type of source, type of overexposure (i.e., local skin, local organ, or global), and documents in which the accident was reported. Reported symptoms, course of treatment, and treatment outcomes were also recorded when available. Finally, accidents were categorized by sector of occurrence: "industrial" including industrial irradiator, production, and radiography; "radiation therapy" including teletherapy, brachytherapy, and therapeutic nuclear medicine; "fluoroscopy" used to support diagnostic and interventional radiology; "military" (e.g. nuclear testing, submarine accidents), and "orphan sources" for overexposures caused by sources fallen outside of regulatory control [19]. A category "others" included overexposure accidents resulting from scientific experiments and unknown causes. For cases with incomplete information, missing data were reported as unknown in our extraction sheet.

### Quality of selected articles and reports

Only cases published in peer-reviewed journals or reported by official experts in radiation management (e.g., IAEA, US NRC, UNSCEAR, REAC/TS) were considered, to ensure the quality of the study. Furthermore, all sources selected for data extraction addressed our review question, which was to understand the characteristics of reported radiation overexposure accidents worldwide and their evolution between 1980 and 2013. Among these, only sources showing evidence of radiation overexposure, as defined in our inclusion criteria, were considered for extraction.

### Analysis

Our extraction sheet was used to assess the distribution of reported radiation overexposure accidents along recorded items and over time.

## Results

### Study selection process

**Fig 1:**
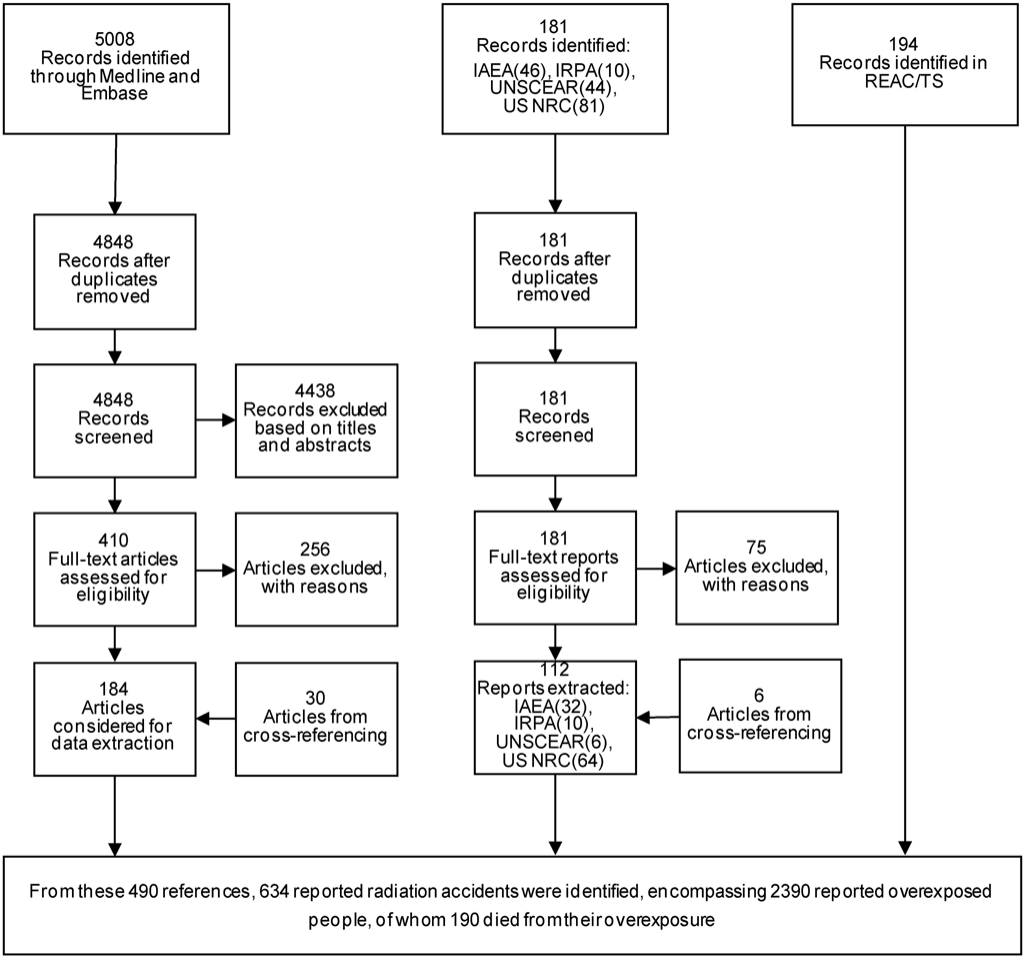
PRISMA flow chart. Search strategy for retrieving reported radiation overexposure accidents worldwide, 1980–2013.

Out of 5189 articles and reports identified, 296 met our eligibility criteria and were extracted (Fig. 1). In addition, 194 records of the REAC/TS radiation accidents registry also met our eligibility criteria and were considered for data extraction. 70 of these 194 records were not reported in any other data source considered for this review. For the period 1980–2013, 634 reported radiation overexposure accidents were identified, encompassing 2390 overexposed people, of whom 190 (8%) died from their overexposure.

Extracted cases were categorized by sector in which the accident occurred and by type of overexposure (Table 1).

**Table 1:** Reported radiation overexposure accidents by sector and type of overexposure worldwide, 1980–2013.

Characteristics of overexposure	Reported accidents	People overexposed	Deaths	References
	n (%)	n (%)	n (%)	
**Industrial**	**169 (27)**	**513 (22)**	**45 (24)**	**-**
Local organ	1	1	0	[20–21]
Local skin	120	158	1	[7–8], [20–69]
Local skin & Global	34	323	35	[7–8], [20–21], [32], [35–36], [39], [46], [55], [69–104]
Global	14	31	9	[7–8], [20–21], [39], [46], [69], [74–75], [82], [105–126]
**Radiation therapy**	**202 (32)**	**1127 (47)**	**96 (51)**	**-**
Local organ	129	407	3	[7–8], [32], [39], [58], [60], [62–66], [100–101], [127–158]
Local skin	61	523	28	[7–8], [20–21], [35–36], [39], [57], [62–63], [66], [100], [127–129], [139], [143], [148], [150], [153–154], [156–179]
Local skin & Local organ	9	182	58	[7–8], [36], [39], [99], [127], [129], [155–156], [164], [175], [180–183]
Local skin & Global	2	13	0	[7–8], [39], [74], [127], [165], [184]
Local organ & Global	1	2	7	[8]
**Fluoroscopy**	**194 (31)**	**400 (17)**	**0 (0)**	**-**
Local organ	41	41	0	[8], [54], [58], [60], [62–65], [100–101], [132], [137], [139], [142], [146], [148–150], [152–153], [156], [170], [172–173], [176], [185–193]
Local skin	152	358	0	[8], [12], [14], [16], [18], [70], [194–263]
Local organ & Global	1	1	0	[61]
**Orphan source**	**32 (5)**	**225 (9)**	**37 (19)**	**-**
Local skin	7	9	0	[8], [20–21], [35], [39], [145], [264–267]
Local skin & Global	20	171	31	[5], [7–8], [20–21], [35–36], [39], [46], [69], [70], [74], [99], [121], [127], [129], [160], [165], [268–286]
Global	5	45	6	[7–8], [35], [39], [69], [99], [127], [129], [287–293]
**Military**	**4 (1)**	**64 (3)**	**12 (6)**	**-**
Local skin	1	1	0	[35]
Local skin & Global	1	59	10	[35], [294–296]
Global	2	4	2	[8], [20–21], [39], [74], [99], [297–299]
**Other** ^**a**^	**33 (5)**	**61 (3)**	**0 (0)**	**-**
Local organ	2	2	0	[8], [20–21], [98]
Local skin	29	57	0	[7–8], [21], [35], [39], [121], [298], [300–302]
Local skin & Global	1	1	0	[35]
Global	1	1	0	[7]
**Total**	**634 (100)**	**2390 (100)**	**190 (100)**	**-**

^a^ Scientific experiments and unknown causes.

### Characteristics of reported radiation overexposure accidents and evolution over time

Among the 634 reported radiation accidents identified, most of them occurred in the industrial sector (27%) and in the medical sector through the use of radiation therapy (32%) or fluoroscopy (31%) (Table 1). Reported accidents in radiation therapy were greater in terms of number of overexposed people (47%), followed by accidents in the industrial sector (22%), fluoroscopy (17%) and orphan sources (9%). Finally, the number of deaths resulting from radiation overexposure, was the greatest for accidents reported in radiation therapy (51%), followed by those reported in the industrial sector (24%) and accidents involving orphan sources (19%).

**Fig 2:**
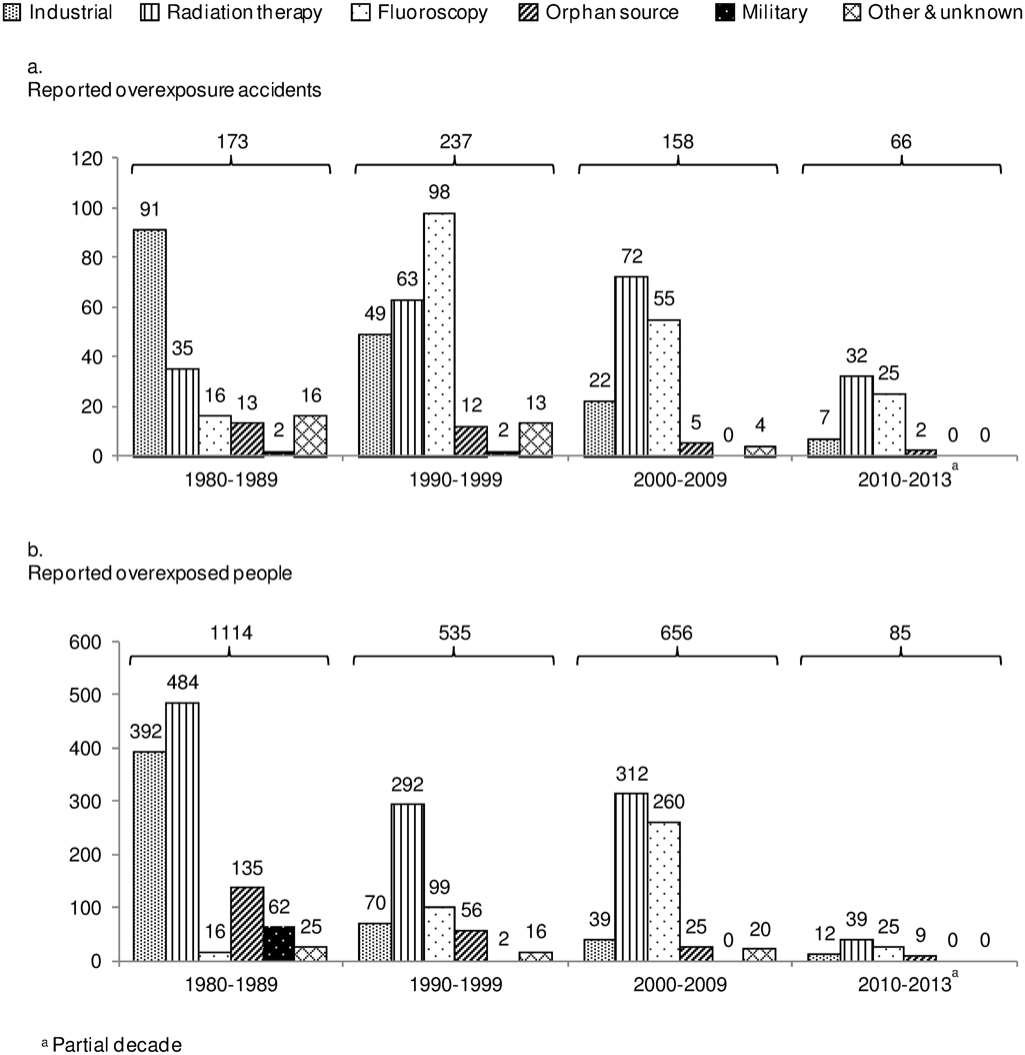
Reported radiation overexposure accidents worldwide and sector involved, 1980–2013. (a) number of reported radiation accidents (b) number of reported overexposed people.^a^Partial decade.

Over the 1980–2009 period, the number of reported radiation accidents and the number of overexposed people by decade exhibited an overall downward trend (Fig. 2a—b). The same trend held for each sector separately, except for accidents reported in radiation therapy and medical fluoroscopy. The number of reported radiation therapy accidents per decade increased along the three decades, however the number of overexposed people experienced an overall decrease within the same period. Moreover, the number of reported fluoroscopic accidents increased significantly from the decade 1980–1989 to 1990–1999. Then, while reported fluoroscopic accidents decreased during the decade 2000–2009, the number of overexposed people increased. While industrial and orphan sources accidents accounted together for most reported accidents between 1980 and 1989 (60%), their proportion sharply decreased afterwards, reaching 17% of reported accidents between 2000 and 2009. Radiation therapy and fluoroscopy accidents experienced the opposite trend and ultimately accounted for most reported accidents and most of overexposed people between 2000 and 2009 (respectively 80% and 87%).

**Fig 3:**
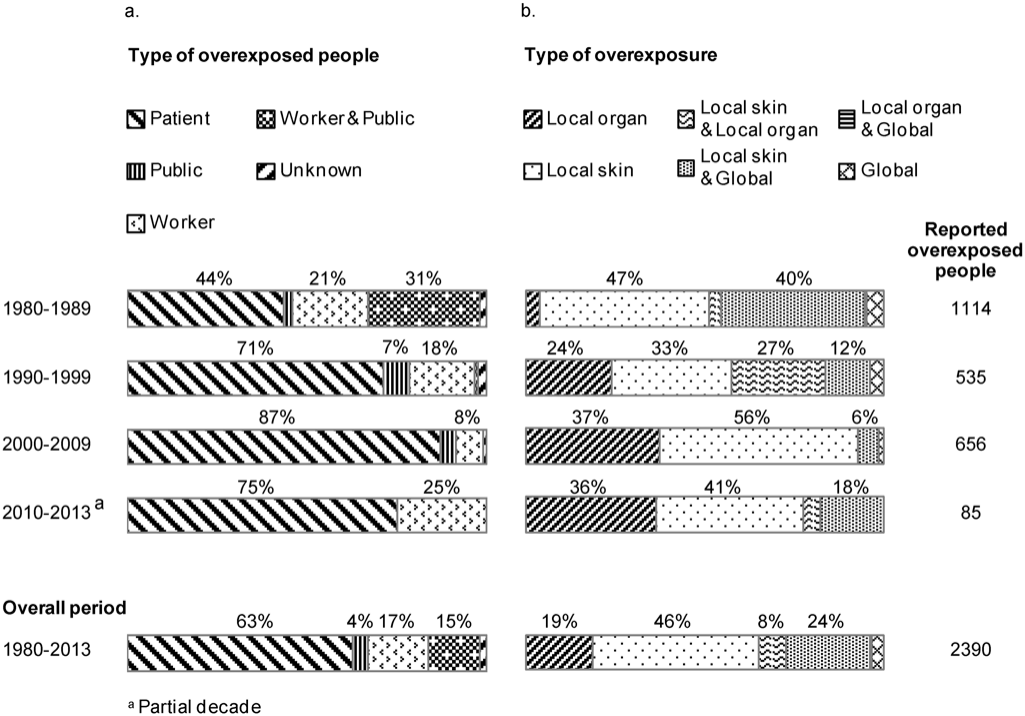
Evolution of type of overexposed people and type of injuries in reported radiation accidents worldwide, 1980–2013. (a) Type of overexposed people (b) Type of overexposure.^a^Partial decade.

Among reported overexposed people, patients represented the largest share (Fig. 3a). Additionally, the share of patients among overexposed people increased along the three decades 1980–1989 (44%), 1990–1999 (71%), and 2000–2009 (87%), while the share of public and workers decreased along the same period.

Finally, the types of overexposure in reported accidents also changed (Fig. 3b). Among retrieved cases, the share of overexposures with a combined local skin and global overexposure decreased from 40% to 6% between the periods 1980–1989 and 2000–2009. The share of global radiation overexposures remained low (below 4%) since the 1980s. However, the share of local organ or local skin overexposures increased over the same period. Local skin overexposures had the highest share during 1980–2013 (46%).

### Profiles of reported radiation overexposures by region

**Fig 4:**
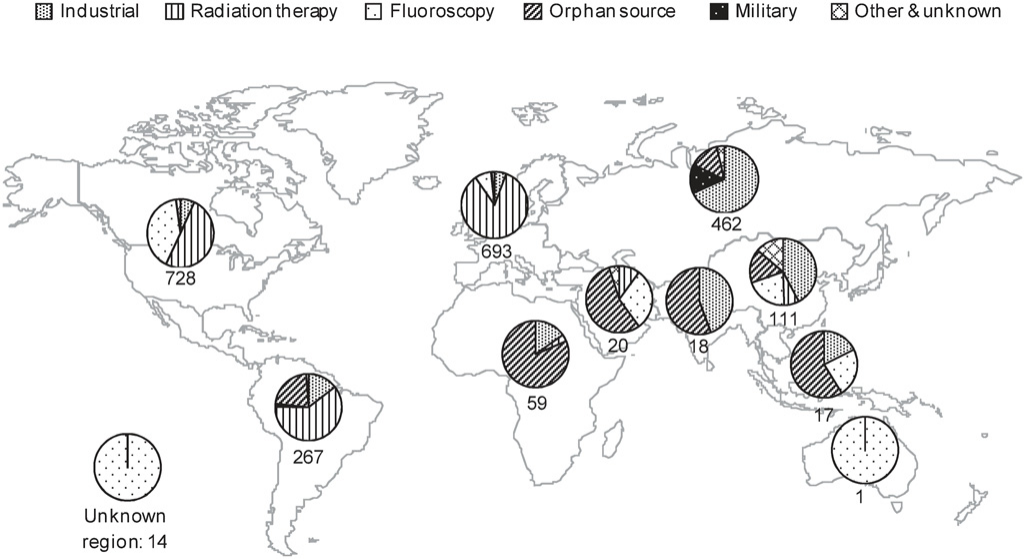
Distribution of sectors involved in reported overexposed people by region, 1980–2013.

The number of cases retrieved varied greatly across geographic regions (Fig. 4). Altogether, North America, South America, Europe and North & Central Asia accounted for 90% of reported overexposures retrieved for the period 1980–2013. Furthermore, the distribution of sectors involved was different from one region to another. Among the four regions with the highest number of reported cases retrieved, the medical sector accounted for most radiation overexposures reported in North America (663 cases, 91%), Europe (642 cases, 93%), and South America (163 cases, 61%). Within the medical sector, the share of reported cases resulting from fluoroscopic overexposures was higher in North America (297 cases, 41%) compared to Europe (55 cases, 8%) and South America (1 case). In contrast, the leading sector of reported radiation overexposure was the industrial one in North and Central Asia (316 cases, 69%).

## Discussion

This review explored a wide array of information sources. It included the assessment of peer-reviewed literature, reports from key national and international organizations in radiation management, and the review of the largest international radiation accidents registry, REAC/TS, for the period 1980–2013.

This review showed that a limited and decreasing number of worldwide radiation accidents have been reported by decade since 1980 and that these accidents can be dramatic, as observed previously [271]. Furthermore, this review suggested that the characteristics of reported radiation overexposure accidents differ over time and across regions. These new findings are important for future interventions in radiation protection.

### Overall downward trends

Our results indicated that the number of reported radiation accidents and overexposed people have decreased over the 1980–2013 period. This likely reflects the impact of continuous efforts in radiation protection. Along the years, the IAEA in conjunction with new ICRP recommendations, has updated safety standards [303]. In 1989, it introduced the Safety Standards Series, which includes Safety Fundamentals, Safety Requirements, and Safety Guides [304]. These standards are used to improve regulation and radiation protection worldwide [305]. For example, ample documentation including lessons learned from accidents in industrial radiography, guidance on safe work practices and training material, have been developed to promote safety prevention in the industrial sector [306–308]. The downward trend in reported radiation accidents could also reflect a decrease in the reporting level. However, this explanation is less likely as the decrease is not observed in all sectors.

### Singularity of the medical sector

This review suggested that the medical sector accounted for most reported radiation overexposures along 1980–2013. Consistently, most reported cases involved patients and a local skin overexposure component.

Unlike other sectors, reported radiation therapy accidents increased along the three last decades, while the number of reported overexposed people tended to decrease. Still, for each decade, radiation therapy accidents represented the highest share of overexposed people.

This upward trend in reported radiation therapy accidents could result from improved reporting or growing use of radiation therapy. UNSCEAR estimated that 5.1 million of radiation therapy treatments were delivered annually worldwide over the 1997–2007 period compared to 4.7 million annually for the period 1991–1996 [158]. The introduction of new technologies (e.g., gamma knife, intensity modulated radiation therapy or IMRT) produced new types of accidental exposures and could also have contributed to this upward trend observed in reported radiation therapy accidents [309–310].

Furthermore, the average number of people involved per reported accident decreased from the 1980’s to the 2000–09 decade. This figure typically varies according to the cause of the accident. While some errors such as errors in treatment site or dose administered, affect a single patient, other errors such as software issue, calibration or treatment programming errors, can affect multiple patients before the issue comes to light. Error reporting systems such as ROSIS allow learning from the past through knowledge of near-misses, incidents or accidents and constitute essential prevention tools [311].

As the use of radiation therapy is expected to grow even more in the future, it is crucial to ensure high quality assurance standards in order to avoid the multiple possible errors in the course of treatment and thus optimize all benefits of radiation therapy [309–311].

This review also brings attention to medical fluoroscopy, which ranked second in number of reported accidents for 1980–2013. Corresponding reported radiation accidents increased significantly from the 1980s to the 1990s, and then decreased. This was consistent with the literature [17–18], [312]. This increase could reflect the expanded use of fluoroscopy since the 1990s. Although fluoroscopy was initially used primarily for diagnostic procedures, it then became widely used during therapeutic interventions (e.g. coronary angioplasty), as it provided a less invasive and costly solution than classic surgery [313]. Potential serious adverse effects of fluoroscopy, however, were rapidly encountered and acknowledged. In 1994, the FDA issued a warning following the reporting of several injuries resulting from the prolonged use of fluoroscopic procedures [312], [314]. Thereafter, the risks of cumulative radiation exposure through fluoroscopy have been documented. Also, important efforts to track and decrease patients' overall exposure to imaging radiation following the ALARA (As Low As Reasonably Achievable) safety principle were initiated worldwide and some programs have been implemented successfully at the sub-national level [315–317].

Of note, the increase in number of reported overexposed people through fluoroscopy from 1990–1999 to 2000–2009 despite the decrease in reported accidents over the same period, is primarily due to a single accident involving 206 patients. Its cause was an error in resetting a CT scan, which went undetected for 18 months [318]. This accident also accounted for about two thirds of reported fluoroscopic overexposure cases in North America.

### Geographic differences

Our exploratory analysis suggests that the causes of reported radiation overexposure accidents differ across regions. One possible explanation relates to variations in radiation equipment and radiation use across countries. The review conducted by UNSCEAR for the period 1991–2007 emphasized that the level of X-ray equipment, radiological examinations, and radiation therapy differ greatly from one country to another and tend to be concentrated in a limited set of countries [158]. For example, during 1997–2007 three-quarters of all radiation therapy treatments were received in countries, which have at least one physician for every 1,000 people in the general population. Still, these countries only represented 24% of the overall surveyed population [158].

Additionally, differences in reporting by country could also account for the observed differences in causes of overexposure. For example, Baeza highlighted the lack of reports from developing countries when going through ROSIS [311].

Thus, these geographic differences should be monitored and accounted for in prevention strategies. Identifying specific needs and practical challenges in the implementation of the safety standards is cornerstone to adjust prevention efforts adequately and efficiently reduce the incidence of radiation injuries.

### Challenges in reporting radiation injuries

The reporting of radiation overexposure injuries faces unique challenges even when a well-established regulation and a solid reporting system network are in place. Indeed, the latency period before the appearance of radiation-related adverse effects varies from days to years [5]. Thus, people can easily go undiagnosed or be misdiagnosed. Furthermore, radiation injuries are uncommon, which can contribute to diagnostic errors [17], [201]. Additionally potential lack of knowledge or access to reporting systems and fear of legal liabilities can be other causes of underreporting [7].

Another major difficulty rises for radiation overexposure accidents resulting from orphan sources or medical fluoroscopy. In these contexts, the exposed subjects often cannot directly relate their injuries to their radiation exposure as they are not aware that they have been exposed to radiation. Even among medical professionals performing procedures assisted by fluoroscopy, a lack of awareness of risks associated with radiation imaging can still exist [209], [244], [319]. Additionally, patients noticing their skin lesions usually seek advice from a dermatologist, but without necessarily providing information about their history of prior fluoroscopy. They might think this information irrelevant or simply forget it. This makes the diagnostic of radiation injury even more challenging for dermatologists [13], [16], [209], [215]. Thus, reported radiation accidents are undoubtedly underestimated.

### Limitations

This review offers a solid basis of reported radiation overexposure accidents to inform radiation protection planning. Still, it has several limitations.

Our literature search only included publications written in English and French languages, which might introduce some publication bias. Moreover, our review was limited to sources of information that were publicly available, with the exception of the REAC/TS registry, which has limited public access. Hence, our review does not capture cases that are exclusively reported in private databases (e.g. hospital registries). Thus, our review likely underestimates the number of reported radiation accidents. Additionally, when the date of overexposure was not reported (125 cases out of 2390), date of first symptoms was used as first proxy (11 cases) and date of report as second proxy (114 cases). This might introduce some bias as symptoms can appear months or years following the overexposure. Finally, reporting country was also used as a proxy for country of occurrence when not mentioned explicitly (116 cases out of 2376 with some localization information).

Despite these limitations, this review captured reported radiation accidents in systematic and consistent way, enabling valuable analysis to support future prevention actions.

## Conclusion

This systematic study updates and broadens the view of reported radiation overexposure accidents. It indicates that reported radiation overexposure accidents are rare and decreased from 1980–1989 to 2000–2009. However, their potential dramatic outcomes stress the importance of radiation protection regulations. This review suggests the greater share of the medical sector in reported overexposures, for which the use of radiation has become central and is expected to grow even more in the future. Thereby, it confirms the importance of quality assurance programs in radiation therapy and medical fluoroscopy.

Finally, this review suggests that the characteristics of reported accidents vary by geography and over time, and are thereby likely to require different interventions. A close reporting and monitoring of radiation overexposure accidents is of great value to inform and prioritize prevention interventions adequately and ultimately reducing further the incidence of these accidents.

## Supporting Information

S1 PRISMA Checklist (PDF)

S1 Protocol (PDF)
