# Supplementary material for: Reported Radiation Overexposure Accidents Worldwide, 1980-2013: A Systematic Review
Source: PLoS One. 2015 Mar 19;10(3):e0118709. doi: 10.1371/journal.pone.0118709 (PMC4366065; doi:10.1371/journal.pone.0118709)
Supplement: S1 Protocol — (PDF) [file pone.0118709.s002.pdf]

SYSTEMATIC REVIEW SEARCH PROTOCOL

Title: Reported radiation overexposure accidents worldwide, 1980-2013: a systematic review

Research Team members:
Dr. Karen Coeytaux, Episight Consulting, Summit, NJ, USA
Prof. Eric Bey, Plastic and Reconstructive Surgery Department, Percy Military Hospital, Clamart, France
Dr. Doran Christensen, DO, Erik S Glassman, Becky Murdock, Radiation Emergency Assistance Center/Training Site (REAC/TS), Oak Ridge, TN, USA
Dr. Christelle Doucet, Celogos, Paris, France

Table of Contents

1. Objective of the study 3
2. Background 3
I. Important characteristics 3
II. Relevance 4
III. Rationale 4
Previous studies 4
Justification for current study 5
3. Methods 5
I. Search strategy 5
II. Selection criteria 6
III. Quality assessment 7
IV. Data extraction 7
V. Data synthesis 7
4. Process 8
I. Resources required to conduct the review 8
II. Dissemination of the findings 8
5. References 8

1. Objective of the study

This study aims at consolidating and completing reviews of reported radiation overexposures currently available. It will systematically review radiation overexposures cases, which have been reported in publically accessible sources (e.g., peer-reviewed literature, governmental reports, reports of international organization in radiation management) and in the Radiation Emergency Assistance Center/Training Site radiation accident registry (REACT/S) registry. Thus, this systematic literature review will inform future prevention and surveillance initiatives.

The study will focus on the last three decades. All retrieved cases will be documented in an excel file along important overexposure accidents characteristics (e.g., dose, source, part of the body overexposed, treatment and follow-up). This data will be analyzed to identify potential trends and made available for future research purposes on this topic.

This study will address the following research questions:

- How has the number of reported radiation overexposures evolved over the last three decades?
- What are the characteristics of reported radiation overexposures (e.g., sector involved, type of injury, type of person involved) and how have they varied overtime?

2. Background

Ionizing radiation overexposure accidents are uncommon, but have a significant burden due to their severe effects and long-term health consequences.

I. Important characteristics

What are the important population and/or disease characteristics (diagnostic criteria, epidemiology, aetiology, prognosis)?

Radiation overexposure accidents have been reported in various sectors where radiation applications are commonly used (e.g., industrial, medical, military sectors) and through the discovery of orphan sources. Thus, population of interest for this study will include workers, patients, and public who have been overexposed in a reported radiation accident (Mettler, 2012).

This review will focus on people presenting deterministic effects induced by radiation overexposure (e.g., acute radiation syndrome, skin radio-dermatitis), as opposed to stochastic effects (e.g. cancer). According to the type of overexposure (e.g., local, global), clinical symptoms will include Acute Radiation Syndrome and Local Radiation Injury. However, the delay between overexposure and injury can vary from weeks to years. Thus, people without clinical symptoms at the time of reporting but exposed to doses acknowledged to induce harmful effects, will be included. Thresholds used have been published in previous studies (see paragraph below on inclusion/exclusion criteria).

II. Relevance

Does the review topic have important implications for health (individual and/or public), as well as health care, policy and research?

The understanding of the epidemiology behind reported radiation overexposures is critical to understand the impact of past prevention efforts and target future ones where they are most needed. It also enables to understand evolutions in types of overexposures and as a result can inform potential needs in adapting treatments and follow-up measures.

Radiation overexposure can have a dramatic impact on people health and require intensive long-term treatment. Treatment needs vary according to the type of overexposure (i.e., local, global). Global overexposure (corresponding to whole body doses of 1 Gray or more) induces acute radiation syndrome characterized by consecutive hematopoietic, gastrointestinal, and neurovascular syndromes. Local skin overexposures of 3 Gy or more and local organ overexposures of 5 Gy or more are likely to lead to acute local radiation injuries (e.g. dermatitis, organ failure), which may be associated with extreme pain. Furthermore, this type of injuries often progresses over time due to inflammatory waves, inducing the spread of radionecrosis (IAEA, 1998).

It is also important from a regulatory and radioprotection perspective to understand the implication and the evolution of the different sectors in which radiation overexposure accidents occur. Indeed, radioprotection efforts and safety guidelines differ by sector and are continuously reinforced and improved along lessons learnt. For example, among others, specific safety guidelines have been developed for the radiation protection and safety in industrial radiography and in radiotherapy (IAEA, 1999; US Nuclear Regulatory Commission, 1998; IAEA, 2006).

III. Rationale

Previous studies

This will be the first systematic review on reported radiation overexposure accidents.

The most extensive review of radiation accidents done at that time was conducted by the United Nations Scientifics Committee on the Effects of Atomic Radiation (UNSCEAR) and includes radiation accidents until July 2007 (UNSCEAR, 2008). UNSCEAR's objectives were to provide "a sound basis for conclusions regarding the number of significant radiation accidents that have occurred, the corresponding levels of radiation exposures and number of deaths and injuries, and the general trends for various practices." However, this review does not consolidate the reported cases in a format that enables to understand trends over time. In addition, it has not been refreshed in the last 6 years and it does not include some types of overexposures such as radiation injuries induced by fluoroscopy. Hence a systematic review should be able to build on and enhance this foundation review reported by UNSCEAR.

Furthermore, some articles present short literature reviews of reported radiation-induced injuries in specific domains. These reviews aim at raising awareness around specific safety issues and/or at sharing knowledge for better diagnosis and treatment. For example, in 1999 Dehen et al. reported two new cases of radiation injury following cardiac catheterisation and reviewed previous overexposures in cardiac interventions (Dehen et al., 1999). In 2012 Boncher et al. described two cases of fluoroscopy-induced injury and listed a dozen of previous publications referring to similar cases (Boncher & Bergfeld, 2012). Although they are informative in their domain, these previous studies are not systematic and do not inform about the evolution of overexposure radiation accidents characteristics over time.

Justification for current study

Conducting a systematic study and documenting cases along radiation overexposure characteristics will consolidate and complete these previous studies, in particular through a clear search and selection strategy, but also thanks to the additional source of REACT/S registry. Thus, it will provide more accurate and up-to-date estimates of reported overexposures, although it is clear that no comprehensive depiction of radiation accidents can be provided due to likely underreporting. It will also provide a view into the potential evolution over time of characteristics of overexposure radiation accidents.

This will serve as a basis to inform the different stakeholders involved in radiation management.

3. Methods

I. Search strategy

Which electronic databases will you search?

Pubmed and Embase

What are your key search terms?

Nuclear, overexposure, accident, radiodermatitis, "radiation dermatitis", radionecrosis, "radiation necrosis", "radiation injury", "radiation injuries", "radiation effect", "radiation effects", "radiation-induced skin", “skin injury", “skin injuries", ulceration, "Acute Radiation Syndrome", Radiotherapy, Computer-Assisted/adverse effects", "Whole-Body Irradiation adverse effects", "Fluoroscopy adverse effects"

What other sources will you search?

International Atomic Energy Agency (IAEA) publications, International Radiation Protection Association (IRPA) congress proceedings, the UNSCEAR reports (1980-2013) and the United States Nuclear Regulatory Commission (US NRC) reports to congress on abnormal occurrences (1980-2012), the REACT/S registry of radiation accidents

II. Selection criteria

The IAEA definition of accident will be used for this study corresponding to "Any unintended event, including operating errors, equipment failures or other mishaps, the consequences or potential consequences of which are not negligible from the point of view of protection or safety" (IAEA, 2014).

What are the inclusion / exclusion criteria?

An overexposed person will be considered as a case if he or she presents at least one of the following criteria:

- Unintended global overexposure of 1 Gy or more
- Unintended local skin overexposure of 3 Gy or more
- Unintended local organ overexposure (e.g. brain, thyroid, prostate) of 5 Gy or more
- Description of clinical presentation providing reasonable index of suspicion for unintended ionizing radiation overexposure (i.e., acute radiation syndrome, radio-dermatitis, permanent alopecia, dry or moist desquamation, blister formation, skin ulceration, dermal atrophy, invasive fibrosis, organ failure, radio-necrosis).

Persons overexposed due to suicide and criminal acts will be excluded.
Dose thresholds have been selected according to previous studies (Aerts et al., 2003; Balter et al., 2010; Garcia Reitbock et al., 2013; ICRP, 2000; ICRP, 2012; Koenig et al., 2001; Mettler, 2012; Otterburn & Losken, 2010; Rehani & Srimahachota, 2011).

Will you impose any additional limits, e.g. language, publication type, study design?

The search will be limited to articles published since January 1st 1980, in the English or French languages.

How will study selection be performed?

First, automatic searches based on key words will be performed in Pubmed and Embase. Duplicates will then be removed. After that, reference titles and summaries will be screened manually. Selected publications will be read in full text for evaluation and extracted if relevant. Finally, cross-referencing and manual search will be used to retrieve additional relevant articles.
For reports from IAEA (nuclear safety reviews, safety reports series, and non serial publications on radiological accidents, IRPA congress proceedings, UNSCEAR reports, and US NRC reports to congress on abnormal occurrences, text will systematically be read in full and extracted if relevant.
For the REACT/S registry, cases will be initially selected based on their date of occurrence (if unknown, date of reporting will be used as a proxy) and based on the reported dose of overexposure (if unknown, case will be selected by default). Selected cases will then be read if full for evaluation and extracted if relevant.
Two independent researchers will screen and review data sources against the inclusion criteria. For selected reports, full-text documents will be evaluated by one reviewer and checked by a second reviewer. Any divergence between reviewers regarding selection process will be resolve through discussion.

III. Quality assessment

Only articles and case reports describing cases published in peer-reviewed journals or reported by official experts in radiation management (e.g., IAEA, US NRC, WHO, UNSCEAR, REAC/TS) will be considered. Among these, only sources showing evidence of radiation overexposure, as defined in our inclusion criteria, will be considered for extraction

IV. Data extraction

What are the key data to be extracted?
Data of interest to be extracted will include:

- Date and place of radiation overexposure accident
- Description of the overexposure accident
- Number of overexposed people
- Number of people dying from their overexposure and time to death
- Type of person involved (i.e., patient, public, or worker)
- Type of overexposure (i.e., global, local skin or local organ)
- Highest global and local dose received in Gray (Gy)
- Type of source involved (e.g., Cobalt 60, Iridium 192, X-ray)
- Sector in which the accident occurred:
    - Industrial: industrial irradiator, production, and radiography
    - Radiotherapy: teletherapy, brachytherapy, and therapeutic nuclear medicine
    - Fluoroscopy (used for diagnostic and interventional radiology)
    - Military
    - Orphan sources
    - Others
- Reported symptoms
- Treatment and treatment outcomes
- Sources in which accident are reported

How will data extraction be performed, and how will extracted data be presented?
Data will be extracted manually and structured in an excel file.

V. Data synthesis

How will data be combined (statistical or narrative), and why?
The count of radiation overexposed people will be presented by characteristics (e.g., sector, type of overexposure, type of person involved) and by decades to present trends of reported radiation overexposures over time. It will also be presented by region with a breakdown along the different sectors for the entire period of the systematic review. No

statistical analysis of time-series will be performed, as events are rare and their number is expected to vary erratically from year to year.

What are the potential sources of effect heterogeneity and how will they be assessed?
N/A

4. Process

I. Resources required to conduct the review

Required expertises for this review include:

- Scientific and medical expertise for the case definition
- Epidemiology expertise for designing/conducting the systematic review

Research databases: Access to Pubmed, Embase, IAEA/UNSCEAR/US NRC reports, the REAC/TS radiation accidents registry.

Bibliographic software: Endnote

II. Dissemination of the findings

Results will be communicated in a peer-reviewed journal to a large audience including environmental health epidemiologists, radiation regulators, experts and clinicians in radiation management. In addition, opportunities to disseminate results in the grey literature will be explored with agencies such as IAEA and UNSCEAR.

5. References

Aerts, A., Decraene, T., van den Oord, J. J., Dens, J., Janssens, S., Guelinckx, P., Garmyn, M. (2003). Chronic radiodermatitis following percutaneous coronary interventions: a report of two cases. *J Eur Acad Dermatol Venereol., 17*(3), 340-343.

Balter, S., Hopewell, J. W., Miller, D. L., Wagner, L. K., & Zelefsky, M. J. (2010). Fluoroscopically guided interventional procedures: a review of radiation effects on patients' skin and hair. *Radiology, 254*(2), 326-341. doi: 10.1148/radiol.2542082312

Boncher, J., & Bergfeld, W. F. (2012). Fluoroscopy-induced chronic radiation dermatitis: a report of two additional cases and a brief review of the literature. *J Cutan Pathol, 39*(1), 63-67. doi: 10.1111/j.1600-0560.2011.01754.x

Dehen, L., Vilmer, C., Humiliere, C., Corcos, T., Pentousis, D., Ollivaud, L., . . . Dubertret, L. (1999). Chronic radiodermatitis following cardiac catheterisation: a report of two cases and a brief review of the literature. *Heart, 81*(3), 308-312.

Garcia Reitbock, J., Feldmann, R., Ruhringer, K., Breier, F., & Steiner, A. (2013). Chronic radiodermatitis following percutaneous transluminal coronary angioplasty. *J Dtsch Dermatol Ges, 11*(3), 265-266. doi: 10.1111/ddg.12004

IAEA. (1998). *Diagnosis and treatment of radiation injuries. Safety Reports Series No. 2*. Vienna: IAEA.

IAEA. (1999). Radiation protection and safety in industrial radiography. Safety reports series No.13. Retrieved from http://www-pub.iaea.org/books/IAEABooks/Series/73/Safety-Reports-Series

IAEA. (2006). *Applying Radiation Safety Standards in Radiotherapy. Safety Reports Series No. 38*. Vienna: IAEA.

IAEA. (2014). Glossary. Retrieved from http://www.iaea.org/ns/tutorials/regcontrol/intro/glossarya.htm

ICRP. (2000). ICRP Publication 85: Avoidance of Radiation Injuries from Medical Interventional Procedures. *Ann. ICRP, 30*(2).

ICRP. (2012). ICRP Publication 118: ICRP statement on tissues and early and late effects of radiation in normal tissues and organs. Threshold doses for tissue reactions in a radiation protection context, 1st Edition Ann. *Ann. ICRP*.

Koenig, T. R., Wolff, D., Mettler, F. A., & Wagner, L. K. (2001). Skin injuries from fluoroscopically guided procedures: part 1, characteristics of radiation injury. *AJR Am J Roentgenol, 177*(1), 3-11. doi: 10.2214/ajr.177.1.1770003

Mettler, F. A. (2012). Medical effects and risks of exposure to ionising radiation. *J Radiol Prot, 32*(1), N9-N13. doi: DOI: 10.1088/0952-4746/32/1/N9

Otterburn, D., & Losken, A. (2010). Iatrogenic fluoroscopy injury to the skin. *Ann Plast Surg, 65*(5), 462-465. doi: 10.1097/SAP.0b013e3181d6e2d3

Rehani, M. M., & Srimahachota, S. (2011). Skin injuries in interventional procedures. *Radiat Prot Dosimetry, 147*(1-2), 8-12. doi: 10.1093/rpd/ncr257

UNSCEAR. (2008). Sources and effects of ionizing radiation. Volume II. Annex C: Radiation exposures in accidents. *Report to the General Assembly*, 1-43.

US Nuclear Regulatory Commission. (1998). Consolidated Guidance About Materials Licenses Program-Specific Guidance About Industrial Radiography Licenses. NUREG-1556. *2*. Retrieved from http://pbadupws.nrc.gov/docs/ML0103/ML010370172.pdf
